# A Novel Few-Shot Learning Framework Based on Diffusion Models for High-Accuracy Sunflower Disease Detection and Classification

**DOI:** 10.3390/plants14030339

**Published:** 2025-01-23

**Authors:** Huachen Zhou, Weixia Li, Pei Li, Yifei Xu, Lin Zhang, Xingyu Zhou, Zihan Zhao, Enqi Li, Chunli Lv

**Affiliations:** 1 China Agricultural University, Beijing 100083, China; 2 Beijing Foreign Studies University, Beijing 100089, China; 3 North China Electric Power University, Beijing 102206, China; 4 Peking University, Beijing 100871, China

**Keywords:** sunflower disease diagnosis, few-shot learning, deep learning in agriculture, attention mechanism

## Abstract

The rapid advancement in smart agriculture has introduced significant challenges, including data scarcity, complex and diverse disease features, and substantial background interference in agricultural scenarios. To address these challenges, a disease detection method based on few-shot learning and diffusion generative models is proposed. By integrating the high-quality feature generation capabilities of diffusion models with the feature extraction advantages of few-shot learning, an end-to-end framework for disease detection has been constructed. The experimental results demonstrate that the proposed method achieves outstanding performance in disease detection tasks. Across comprehensive experiments, the model achieved scores of 0.94, 0.92, 0.93, and 0.92 in precision, recall, accuracy, and mean average precision (mAP@75), respectively, significantly outperforming other comparative models. Furthermore, the incorporation of attention mechanisms effectively enhanced the quality of disease feature representations and improved the model’s ability to capture fine-grained features.

## 1. Introduction

Bayannur, located in northern China, is an important agricultural production base and is renowned for its high-quality sunflower cultivation [1]. Sunflowers not only constitute a crucial component of the local economy but also serve as a significant source of income for farmers [2,3]. However, in recent years, diseases have posed a severe threat to sunflower growth and yield. Diseases such as downy mildew, rust, and sclerotinia often occur in the Bayannur region [4]. These diseases typically appear abruptly and exhibit regional and seasonal characteristics, making traditional manual monitoring methods ineffective in timely detection and control. The ongoing spread of these diseases not only impacts farmers’ economic returns but also presents a potential threat to the sustainable development of agricultural ecosystems [5,6]. Therefore, the development of precise disease detection technologies for sunflowers holds significant practical value [7,8]. Accurate disease detection not only helps agricultural practitioners to identify and address issues in a timely manner but also reduces the ineffective use of pesticides, thereby minimizing ecological pollution [9]. Moreover, with the advancement in agricultural digitization, intelligent disease detection systems have become a key focus of modern agricultural development [10]. Particularly in large-scale cultivation areas like Bayannur, deploying efficient and convenient disease detection technologies can significantly enhance agricultural productivity, improve crop quality and yield, and promote the modernization of local agriculture [11,12].

The traditional methods for sunflower disease monitoring are primarily based on manual observation, with agricultural technicians or farmers relying on their experience to identify diseases. While this method offers flexibility and low costs, it has clear limitations [13,14]. First, manual disease identification is subjective and prone to errors due to individual judgment. Second, manual monitoring in large-scale fields is inefficient, especially when early symptoms of diseases are not apparent, making it easy to miss the optimal control window. Finally, manual monitoring is not suited to meet the practical needs of large environmentally complex agricultural regions such as Bayannur. In recent years, image-processing-based disease detection methods have gradually been applied in agriculture [15]. These methods typically rely on traditional feature extraction and machine learning techniques, such as Support Vector Machine (SVM) [16] and random forest (RF) [17], utilizing plant leaf features such as color, texture, and shape for disease classification. However, these methods require extensive manual feature design and perform poorly under complex backgrounds (e.g., interference from weeds, soil, etc.). Moreover, traditional image processing methods have limited performance when dealing with small-sample data, making them unsuitable for the diversity and uncertainty encountered in real-world agricultural production.

The occurrence of sunflower diseases is characterized by rapid spread and widespread impact, and these diseases are often difficult to detect in their early stages. Once the diseases spread, they can cause irreversible damage to crop yield. Therefore, timely detection and control are critical to ensuring the healthy growth of crops. This is especially true for primary agricultural regions like Bayannur, where disease monitoring and management are of strategic importance to agricultural production safety [18]. The limitations of the traditional disease monitoring methods have led researchers to focus on automated disease detection technologies. Automated techniques not only significantly enhance disease detection efficiency but also provide more accurate results by analyzing large-scale datasets [8]. However, the complexity of agricultural environments and the diversity of disease types present numerous challenges in the development of disease detection technologies [14]. These challenges include scarce sample data, optimization of small-sample problems, and disease feature extraction under multiple background interference conditions. Therefore, the development of disease detection technologies that are capable of efficiently handling small-sample scenarios is of paramount importance for improving agricultural productivity and advancing agricultural modernization [12,19].

With the advancement in deep learning technologies, convolutional neural network (CNN)-based disease detection methods have attracted increasing attention in agriculture. These methods enable efficient disease recognition and classification by training models on large-scale datasets [19,20]. In object detection tasks, methods such as Faster R-CNN, YOLO, and DETR have been widely applied and have achieved remarkable results across various fields [3]. However, deep learning methods typically require vast amounts of annotated data for training, whereas agricultural disease data are often scarce and imbalanced, limiting the direct application of traditional deep learning methods in agricultural disease detection [21]. Gulzar et al. trained and evaluated five widely used deep learning models—AlexNet, VGG16, InceptionV3, MobileNetV3, and EfficientNet—using a sunflower disease image dataset. The results showed that EfficientNetB3 achieved the highest accuracy, recall, F1-score, and accuracy rate, all reaching 0.979, while the other models (AlexNet, VGG16, InceptionV3, and MobileNetV3) achieved accuracy rates of 0.865, 0.965, 0.954, and 0.969, respectively [22]. Rajbongshi et al. proposed a sunflower disease recognition method where k-means clustering was used to segment the disease-affected areas after image processing. Features were then extracted from the segmented images, and classification was performed using five classifiers. The random forest classifier achieved the highest average accuracy of 0.91, outperforming the other classifiers [10]. Malik et al. introduced a hybrid deep learning model for sunflower disease classification, which employed stacked ensemble learning and combined two models—VGG-16 and MobileNet—achieving an accuracy of 0.89 [23]. Sathi et al. proposed a strategy for sunflower disease recognition where k-means clustering was used to segment the disease-affected areas after image processing, followed by feature retrieval from these areas. Four deep learning classifiers were employed for classification, with the ResNet50 classifier showing the best performance, with an average accuracy of 0.98, while Inception V3 had the lowest accuracy [20,24].

First, the traditional methods struggle to adapt to the complexity and diversity of disease features. Second, the reliance of deep learning methods on large-scale labeled data limits their application in small-sample scenarios. Third, the existing data augmentation and transfer learning methods are insufficiently adaptable to scenarios with complex backgrounds and rare diseases. Fourth, the application of generative models, particularly diffusion models, in agricultural disease detection remains underexplored, with a lack of studies integrating theory and practice. To address the sunflower disease detection needs in Bayannur, this study proposes a detection method based on few-shot learning and diffusion models and successfully implements the model deployment in real-world scenarios. The specific innovations are as follows:

1. Introduction of diffusion models: For the first time, a diffusion model is applied to sunflower disease detection, enhancing the model’s performance in small-sample scenarios by generating high-quality augmented samples.
2. Few-shot learning framework: A few-shot-learning-based disease detection network is designed, combining an attention mechanism to enhance the extraction of features from critical disease areas.
3. Customized loss function: A new diffusion loss function is proposed, tailored to the generation characteristics of diffusion models, significantly improving the quality of the generated data and the stability of the model.
4. Real-world deployment: The proposed method is validated not only in laboratory environments but also through successful deployment in agricultural practices in Bayannur, providing direct technical support for local agricultural production.

In conclusion, the method proposed in this study offers new insights for sunflower disease detection and explores new directions for the application of few-shot learning and diffusion models. Through validation in real-world scenarios, the practicality and potential for the widespread adoption of this method in agriculture are demonstrated.

## 2. Related Work

Few-shot learning (FSL) [25], Generative Adversarial Networks (GANs) [26], and diffusion models [27] are the three primary methods that are currently employed to address the issue of data scarcity in few-shot scenarios. These methods have demonstrated exceptional performance in image classification, object detection, and generative tasks, providing significant support for solving practical problems. This section discusses the principles and applications of these methods in the context of sunflower disease detection, incorporating relevant computational formulas to illustrate their mathematical foundations.

### 2.1. Few-Shot Learning

Few-shot learning aims to solve the model generalization problem in scenarios where training samples are limited. The core idea is to quickly learn effective feature representations from a small number of samples using techniques such as meta-learning or transfer learning [28]. The typical steps of few-shot learning include task representation, support set training, and fast adaptation to the test set [29]. During the training process, the model learns from a set of tasks $T=T_{1},T_{2},\ldots ,T_{n}$, where each task contains a small support set S and a query set Q. The goal is to maximize the log-likelihood of the task

(1) $$L_{FSL}=\sum_{i=1}^{n}\log P\left(Q_{i}|S_{i};\theta \right),$$

where θ represents the model parameters, and $P(Q_{i}|S_{i};\theta )$ denotes the prediction probability for the query set $Q_{i}$ given the support set $S_{i}$. One important implementation of few-shot learning is the metric-based approach, such as prototypical networks. In this method, the feature center $c_{k}$ for each class is computed, and classification is performed based on the Euclidean distance between the feature and the center

(2) $$c_{k}=\frac{1}{|S_{k}|}\sum_{x\in S_{k}}f_{\theta }\left(x\right),\;P\left(y=k|x\right)=\frac{\exp(-d\left(f_{\theta }\left(x\right),c_{k}\right))}{\sum_{j}\exp\left(-d\left(f_{\theta }\left(x\right),c_{j}\right)\right)},$$

where $f_{\theta }\left(x\right)$ is the embedding representation of sample x, and d(·,·) represents the distance function. In this formula, *y* represents the predicted class label for the input x, where y∈{1,2,⋯,K} corresponds to one of the *K* possible disease categories. The probability P(y=k|x) is the likelihood of the input x belonging to the class *k*, calculated based on the distance between the feature representation $f_{\theta }\left(x\right)$ and the prototype $c_{k}$ for class *k*. For the task of sunflower disease detection, this method enables efficient classification by effectively extracting disease features, even in scenarios with scarce samples.

### 2.2. Generative Adversarial Networks (GANs)

GANs are generative models that generate high-quality sample data through the adversarial game between a generator and a discriminator [30]. The generator is responsible for producing realistic samples from random noise, while the discriminator evaluates whether the input sample is real or generated [31]. The goal of a GAN is to make the generated samples increasingly indistinguishable from real samples. Its loss function is provided by

(3) $$L_{GAN}=E_{x∼p_{data}\left(x\right)}\left[\log D\left(x\right)\right]+E_{z∼p_{z}\left(z\right)}\left[\log\left(1-D\left(G\left(z\right)\right)\right)\right],$$

where D(x) is the output probability of the discriminator for a real sample x, G(z) is the sample generated by the generator from noise z, and $p_{data}\left(x\right)$ and $p_{z}\left(z\right)$ represent the real data distribution and the noise distribution, respectively. In this formula, E represents the expected value, or expectation, of the given expression under the specified probability distribution. GANs have demonstrated remarkable performance in image generation and data augmentation tasks, making them effective for expanding small-sample datasets. However, GANs require large amounts of data for training to ensure high-quality generation and are prone to mode collapse, where the generator produces limited sample patterns with low diversity [32]. For sunflower disease detection, GANs can generate images of various disease manifestations to augment the dataset, but their training instability may affect the reliability of the generated results [33].

### 2.3. Diffusion Models

Diffusion models are generative methods based on probabilistic models that generate samples by gradually perturbing data distributions into Gaussian distributions and reversing the process to restore the data distribution [34]. The core idea of diffusion models is to define a forward process and a reverse process, where the data distribution is gradually transformed from a complex form into a simple distribution, and then realistic data samples are generated in reverse [35]. The forward diffusion process gradually adds noise to the data and is defined as

(4) $$q\left(x_{t}|x_{t-1}\right)=N\left(x_{t};\sqrt{1-\beta_{t}}x_{t-1},\beta_{t}I\right),$$

where $x_{t}$ represents the sample at the *t*-th step, and $\beta_{t}$ denotes the noise intensity. Through multiple steps, the data are gradually transformed into pure Gaussian noise. The reverse process learns a parameterized denoising distribution to recover the data

(5) $$p_{\theta }\left(x_{t-1}|x_{t}\right)=N\left(x_{t-1};\mu_{\theta }\left(x_{t},t\right),\Sigma_{\theta }\left(x_{t},t\right)\right),$$

where $\mu_{\theta }\left(x_{t},t\right)$ and $\Sigma_{\theta }\left(x_{t},t\right)$ represent the parameterized denoising mean and variance. The overall goal of diffusion models is to maximize the log-likelihood estimate of the entire process

(6) $$L_{Diffusion}=E_{q(x_{0:T})}\left[\sum_{t=1}^{T}D_{KL}\left(q\left(x_{t-1}|x_{t},x_{0}\right)∥p_{\theta }\left(x_{t-1}|x_{t}\right)\right)\right],$$

where $D_{KL}$ denotes the Kullback–Leibler divergence, which measures the difference between two distributions. Diffusion models exhibit high stability and generation quality in generative tasks, making them particularly suitable for small-sample scenarios [36]. For sunflower disease detection, diffusion models not only generate diverse disease data but also ensure the effectiveness of data augmentation due to the stability of the generation process [37]. In this study, the fast adaptation capability of few-shot learning, the data augmentation advantage of GANs, and the high-quality generation capability of diffusion models are integrated into a novel disease detection framework. The diverse augmented data generated by the diffusion model enhance the variety of the sunflower disease detection dataset, while few-shot learning’s feature learning ability addresses the generalization problem in small-sample data. Additionally, a comparison of the generation effects between GANs and diffusion models was conducted in experiments, demonstrating the advantages of diffusion models in data augmentation for disease detection. This combined approach holds significant practical implications for deployment in the Bayannur region. By generating diverse sunflower disease images, the robustness and generalization ability of detection models are enhanced, providing efficient and reliable technical support for local agricultural production.

## 3. Materials and Methods

### 3.1. Dataset Collection

**Figure 1:**
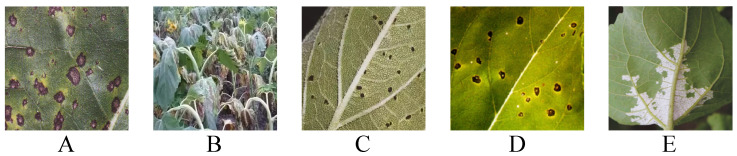
Dateset samples: (**A**) is brown spot disease; (**B**) is wilt disease; (**C**) is rust disease; (**D**) is black spot disease; (**E**) is downy mildew.

The dataset collection for this study primarily focused on common sunflower diseases, including black spot disease, brown spot disease, downy mildew, wilt disease, and rust disease, as shown in Figure 1. The images in the dataset were collected from Bayannur City in Inner Mongolia and supplemented with images from online sources from March to October in 2023–2024, aiming to provide a diverse and representative sample library for model training and evaluation. The number of images for each disease ranged from 1000 to 2000, ensuring sufficient and diverse samples for each disease, thus supporting the model’s generalization ability, as summarized in Table 1.

The equipment and technical requirements for image collection were high to ensure that the collected images met the standards required for deep learning model training. Therefore, the collection process included high-resolution digital cameras and related auxiliary equipment, such as tripods, stabilizers, and light sources. The primary digital camera used was a high-pixel device (e.g., Canon EOS 5D Mark IV), capable of providing clear and detailed images to capture the subtle features of the diseases. Lens selection was also critical, with macro lenses chosen to precisely capture the fine details of the sunflower leaves and lesions. Additionally, controlling the lighting was crucial as both excessive and insufficient lighting would affect the image quality, particularly in terms of presenting details. Therefore, a combination of natural light and additional lighting was typically used to ensure even illumination, making the lesions clearly visible. To avoid interference from external environmental factors, image collection was generally performed under clear weather conditions, avoiding overcast days or conditions with variable lighting. During the data collection process, strict attention was paid to the details of each image. Factors such as the shooting angle, distance, light intensity, and focal length of each image were carefully designed to ensure high consistency and comparability of images across different disease types. For example, in the collection of images for black spot disease, special attention was paid to capturing the shape, color, and contrast between the lesions and healthy areas, ensuring that the model could learn to recognize the key features of the lesions. Black spot lesions are typically black or dark brown with irregular edges and generally have a diameter ranging from 1 to 3 centimeters. Therefore, the focus during image collection was on the contour and distribution characteristics of the lesions to facilitate subsequent disease classification and detection.

For brown spot disease, particular attention was paid to the color variations in the lesions, which are typically dark brown or purple, sometimes with a yellow ring in the center, and more regular edges. Thus, the image collection emphasized the color contrast between the lesion center and edges to help the model distinguish between different disease types. Downy mildew, characterized by white fungal growth on the undersides of leaves, required careful attention to the distribution of mildew on the leaf surface, ensuring that these features were accurately captured. Wilt disease primarily manifests as the gradual yellowing and wilting of the leaves. During image collection, particular care was taken to ensure a distinct contrast between the wilted areas and the healthy regions of the leaves to facilitate model training and differentiation. Rust disease is identified by the formation of rust-colored lesions on the leaf surface. During image capture, meticulous attention was paid to the shape, distribution, and color of the lesions to ensure accurate identification. In addition to the field collection from Bayannur City, part of the dataset was supplemented with network images. The collection of network images primarily relied on search engines and agricultural disease image libraries, ensuring that sunflower disease images from different regions and climatic conditions were included. The advantage of network images lies in their diversity as they provide rare disease samples that are difficult to obtain in field collection, as well as representations of diseases at different growth stages. To ensure the quality of the network images, each image was rigorously screened to exclude low-quality, blurry, or unrelated images. These network images, together with the field-collected images, provided a rich set of training data for the model.

### 3.2. Data Augmentation

Data augmentation is a widely used technique in deep learning tasks to enhance the model’s generalization ability. Traditional augmentation methods, such as rotation, flipping, and color perturbations, are simple and effective; however, they may not provide sufficient diversity in training samples for some complex scenarios. To address the specific needs of sunflower disease detection, some less common but highly effective augmentation techniques, such as MixUp and CutMix, are employed. These methods, combined with the advantages of generative models, improve the diversity and robustness of the dataset by generating pseudo-samples.

#### 3.2.1. MixUp

MixUp is a simple yet effective data augmentation technique based on the idea of linear interpolation, where two images and their corresponding labels are mixed in a specific ratio to generate new samples. Specifically, for any two input images $x_{i}$ and $x_{j}$, and their corresponding labels $y_{i}$ and $y_{j}$, the augmented sample $\tilde{x}$ and $\tilde{y}$ are defined as follows:

(7) $$\tilde{x}=\lambda x_{i}+\left(1-\lambda \right)x_{j},\;\tilde{y}=\lambda y_{i}+\left(1-\lambda \right)y_{j},$$

where λ is a random number sampled from the interval [0,1], typically following a Beta distribution, i.e., λ∼Beta(α,α), where α>0 is a hyperparameter controlling the degree of mixing. This linear interpolation approach not only generates more diverse samples in the image space but also introduces smooth transitions in the label space. This smooth label mechanism effectively mitigates overfitting and enhances the model’s learning capability for boundary samples. In the sunflower disease detection task, the use of MixUp can simulate complex disease distribution characteristics under different environmental conditions. For example, mixing images of mildly infected plants with healthy plant images generates samples with varying degrees of disease, thereby improving the model’s ability to distinguish boundary diseases.

#### 3.2.2. CutMix

CutMix is a more aggressive data augmentation technique. Unlike MixUp, CutMix directly replaces a region of one image with the corresponding region from another image. For any two images $x_{i}$ and $x_{j}$, and their corresponding labels $y_{i}$ and $y_{j}$, the augmented sample is defined as follows:

(8) $$\tilde{x}=M⊙x_{i}+\left(1-M\right)⊙x_{j},\;\tilde{y}=\lambda y_{i}+\left(1-\lambda \right)y_{j},$$

the symbol ⊙ represents the element-wise (Hadamard) product, where M is a binary mask matrix that determines the fusion pattern of images $x_{i}$ and $x_{j}$, and λ represents the area ratio of the replaced region, computed as

(9) $$\lambda =\frac{\mathrm{Area}(M)}{\mathrm{Total}\;\mathrm{Area}}.$$

The advantage of CutMix lies in its direct enhancement of the model’s focus on local features by replacing part of an image with another. This is particularly important in sunflower disease detection tasks as diseases are typically concentrated in localized regions of the leaves. By applying CutMix, augmented samples with mixed disease features can be generated, enabling the model to better learn complex local disease patterns.

#### 3.2.3. Combination of MixUp and CutMix

To further enhance the data augmentation effect, the MixUp and CutMix methods are combined. This approach strikes a good balance between global and local augmentation. For example, for a sunflower disease image $x_{i}$ and a healthy plant image $x_{j}$, a linearly mixed sample $x_{mix}$ can first be generated using MixUp, and then a portion of the region can be replaced with a block from another disease image $x_{k}$ using CutMix. The mathematical representation is as follows:

(10) $$\tilde{x}=M⊙\left(\lambda x_{i}+\left(1-\lambda \right)x_{j}\right)+\left(1-M\right)⊙x_{k}.$$

the symbol ⊙ represents the element-wise (Hadamard) product. This multi-level augmentation approach not only increases the diversity of the data but also preserves the feature distribution of different disease types.

#### 3.2.4. Diffusion-Model-Assisted Data Augmentation

In addition to MixUp and CutMix, a diffusion model is employed to generate high-quality pseudo-samples to further enrich the dataset. The generation process of a diffusion model can be viewed as a reverse process of progressively restoring a simple distribution (such as a Gaussian distribution) to the complex data distribution. Specifically, in the diffusion model, sample generation follows the reverse conditional distribution

(11) $$p_{\theta }\left(x_{t-1}|x_{t}\right)=N\left(x_{t-1};\mu_{\theta }\left(x_{t},t\right),\Sigma_{\theta }\left(x_{t},t\right)\right),$$

where $\mu_{\theta }\left(x_{t},t\right)$ and $\Sigma_{\theta }\left(x_{t},t\right)$ are the model’s parameterized denoising mean and variance. By inputting real disease images as initial samples into the diffusion model, pseudo-samples with disease features but complex backgrounds can be generated. These samples not only enhance the diversity of the data but also provide additional boundary sample information, further improving detection performance. MixUp and CutMix, through different data augmentation mechanisms, enhance the model’s perception of disease features from the perspectives of global feature fusion and local feature mixing. Meanwhile, the diffusion model generates high-quality pseudo-samples, providing more representative samples for the dataset. The combination of these three methods demonstrates significant advantages in sunflower disease detection tasks, particularly in data-scarce scenarios, effectively enhancing the model’s robustness and generalization ability. By employing these less common data processing techniques, various disease distribution characteristics under different degrees and environmental conditions have been successfully simulated, overcoming the limitations of traditional augmentation methods. This provides solid technical support for sunflower disease detection in the Bayannur region.

Sunflower disease detection tasks involve highly diverse and complex features as disease symptoms often vary significantly in terms of color, shape, and size across different samples. In addition, background interference in field environments is a significant challenge, where disease regions often exhibit low contrast with healthy areas, and certain non-disease regions may display pseudo-features resembling disease traits. Furthermore, the issue of data scarcity is particularly pronounced, especially in early disease stages or rare disease types, where annotated data are insufficient to provide comprehensive training support for deep learning models. To address these challenges, MixUp, CutMix, and diffusion models were selected due to their ability to enhance feature diversity and robustness in this specific context. MixUp generates “blended-feature” samples by linearly combining pairs of images and their corresponding labels, effectively covering a broader range of feature spaces for disease characteristics. This improves the model’s ability to generalize across diverse disease traits and better adapt to unseen data. Meanwhile, CutMix is particularly effective in addressing the detection of small and unevenly distributed disease regions. For example, in cases such as black spot disease or rust disease, where lesions are often small and highly localized, CutMix creates synthetic samples by replacing portions of an image with patches from another. This enables the model to focus more effectively on small but critical disease regions, thereby enhancing its detection performance. The incorporation of diffusion models further strengthens the augmentation process by addressing both the data scarcity problem and the complexity of field environments. Diffusion models generate synthetic samples with diverse backgrounds, disease stages, and morphological variations, providing the model with enriched training data that mimic real-world variability. This capability not only improves the model’s robustness to complex and noisy backgrounds but also ensures better adaptability to rare and underrepresented disease types. These carefully chosen augmentation methods collectively contribute to enhancing the performance and reliability of the proposed framework in addressing the specific challenges of sunflower disease detection.

### 3.3. Proposed Method

**Figure 2:**
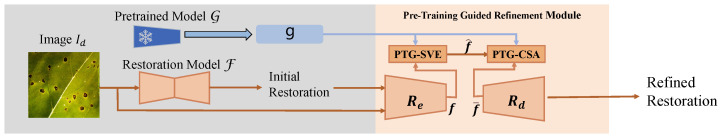
Flowchart of the disease feature refinement and recovery module guided by the pre-trained model.

The proposed method consists of multiple interconnected modules that process input data through a sequence of initial restoration, feature generation, and enhancement guided by a pre-trained model, and refined disease detection, forming an end-to-end detection system, as shown in Figure 2. The symbol $\bar{f}$ represents the feature enhancement result guided by the pre-trained model, specifically generated by the PTG-SVE (Pre-Training Guided Spatial Variance Enhancement) module. The PTG-SVE module performs dynamic optimization of spatial variance by combining the global feature *g* generated by the pre-trained model *G* with the input initial feature *f*, thus highlighting the feature representation of the disease region while suppressing background noise interference. The mathematical expression of this process is

(12) $$\bar{f}=\mathrm{PTG}-\mathrm{SVE}\left(f,g\right),$$

where *f* is the initial feature generated by the initial recovery model F. The main function of the initial recovery model F is to extract the preliminary feature *f* from the input disease image $I_{d}$, providing input for the subsequent modules as follows:

(13) $$f=F(I_{d}).$$

Subsequently, the enhanced feature $\bar{f}$ is passed to the PTG-CSA (Pre-Training Guided Contextual Self-Attention) module, which globally models the features using a contextual self-attention mechanism. The goal of the PTG-CSA module is to capture the complex dependencies between the disease region and the background, generating the optimized feature $\hat{f}$, expressed mathematically as

(14) $$\hat{f}=\mathrm{PTG}-\mathrm{CSA}\left(\bar{f}\right).$$

Next, the feature $\hat{f}$ is sequentially input into the recovery enhancement module $R_{e}$ and the recovery refinement module $R_{d}$ to further refine the disease features and generate the final detection result. The recovery enhancement module $R_{e}$ performs local optimization on the features to make them more suitable for the feature representation of the disease region, while the recovery refinement module $R_{d}$ completes the high-precision localization and classification of the disease region

(15) $$f_{e}=R_{e}\left(\hat{f}\right),\;f_{d}=R_{d}\left(f_{e}\right),$$

where $f_{e}$ is the enhanced feature, and $f_{d}$ is the final recovered feature. The revision also includes a clear description of the workflow of the entire module. The input image $I_{d}$ is first processed by the initial recovery model F to extract the basic feature *f*, which is then optimized for variance by the PTG-SVE module combined with the global feature *g* generated by the pre-trained model *G*, resulting in the enhanced feature $\bar{f}$. Next, $\bar{f}$ is input into the PTG-CSA module, where contextual modeling generates the optimized feature $\hat{f}$. Finally, $\hat{f}$ sequentially passes through the recovery enhancement module $R_{e}$ and the recovery refinement module $R_{d}$ to produce the final disease detection result.

#### 3.3.1. Few-Shot Diffusion Detection Network

**Figure 3:**
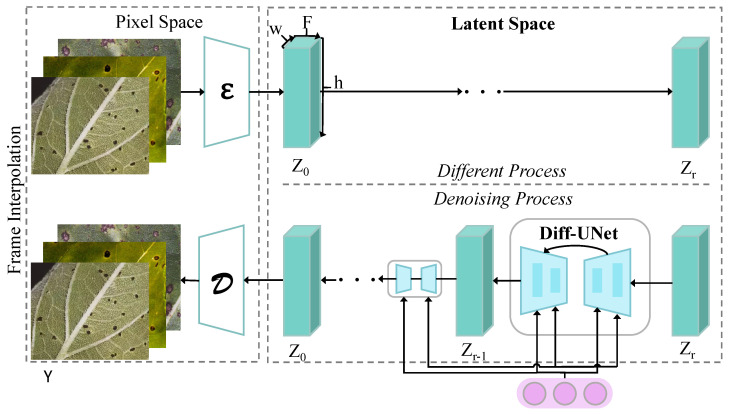
Flowchart of the disease detection method based on the diffusion model, illustrating pixel space, latent space, and key modules for generation and denoising processes.

The few-shot diffusion detection network (FSDDN) combines the efficient feature learning capability of few-shot learning with the high-quality data generation capability of diffusion models, addressing the challenges of limited data and insufficient generalization in disease detection tasks. The architecture of this network, as shown in Figure 3, is built upon a diffusion generative model framework. It generates high-quality disease image features through a specific denoising process and integrates these features with a few-shot learning module to achieve high-precision detection and classification of disease regions.

In the FSDDN, the input image is first mapped to a latent space, forming the initial feature representation $Z_{0}$. This mapping process is performed by an encoder ϵ, defined as

(16) $$Z_{0}=\epsilon \left(I_{d}\right),$$

where $I_{d}$ is the input disease image, and ϵ is a deep convolutional network responsible for extracting global and local features from the pixel space. The latent feature $Z_{0}$ then enters the diffusion process, where Gaussian noise is progressively added to simulate complex distributions. The forward formula of the diffusion process is provided by

(17) $$q\left(Z_{t}|Z_{t-1}\right)=N\left(Z_{t};\sqrt{1-\beta_{t}}Z_{t-1},\beta_{t}I\right),$$

where $Z_{t}$ represents the feature at step *t*, and $\beta_{t}$ denotes the noise strength parameter. At each step, the features deviate further from their original distribution, eventually reaching a completely random Gaussian distribution $Z_{r}$. To recover disease features from the noisy distribution, the FSDDN employs a denoising process utilizing a parameterized diffusion inversion network (Diff-UNet) to iteratively restore $Z_{r}$ to $Z_{0}$. The reverse formula of the denoising process is

(18) $$p_{\theta }\left(Z_{t-1}|Z_{t}\right)=N\left(Z_{t-1};\mu_{\theta }\left(Z_{t},t\right),\Sigma_{\theta }\left(Z_{t},t\right)\right),$$

where $\mu_{\theta }\left(Z_{t},t\right)$ and $\Sigma_{\theta }\left(Z_{t},t\right)$ are the predicted mean and variance, computed by Diff-UNet. Diff-UNet adopts a U-Net architecture with multi-scale feature fusion and skip connections, effectively capturing the complex features of disease regions. At each denoising step, Diff-UNet leverages contextual self-attention modules to capture global relationships while extracting local detail features using convolution operations. After completing the diffusion process, the generated features ${\hat{Z}}_{0}$ are input into the few-shot learning module for classification and detection. In this module, category prototypes are learned using prototypical networks, where the category prototype is defined as

(19) $$Z^{\prime }\left(i,j,k\right)=\sum_{c=1}^{C}W\left(c,k\right)\cdot Z\left(c,i,j\right),$$

Here, Z is the input feature tensor with dimensions (C,H,W), W is the weight matrix with dimensions (C,K), and $Z^{\prime }$ is the output tensor with dimensions (K,H,W). This formula clarifies the weighted summation process along the channel dimension while eliminating any potential ambiguity or vagueness. The prediction for a query sample is determined by the distance to the category prototype as follows:

(20) $$P\left(y=k|x\right)=\frac{\exp(-d\left(f_{\theta }\left(x\right),c_{k}\right))}{\sum_{j}\exp\left(-d\left(f_{\theta }\left(x\right),c_{j}\right)\right)}.$$

The network configuration specifies that the encoder ϵ and the convolutional layers in Diff-UNet are implemented using ResNet-50 and a modified U-Net structure, respectively, with channel dimensions of 64, 128, 256, and 512 for each layer to accommodate multi-scale feature extraction. The number of diffusion steps *T* is set to 1000, ensuring the generation of high-quality latent feature distributions while maintaining computational efficiency. This design offers significant advantages for the task at hand. First, the diffusion process generates latent features that compensate for the imbalances and insufficiencies in small-sample data, enhancing feature diversity. Second, the multi-scale feature fusion design of Diff-UNet ensures precise capture of disease regions even in complex backgrounds, particularly for modeling fine-grained disease features. Finally, the incorporation of the few-shot learning module facilitates efficient classification of disease categories by learning category prototypes, ensuring robust detection performance even with limited data. The synergy of mathematical formulations and network architecture enables this approach to achieve exceptional generalization and detection performance in complex agricultural scenarios.

#### 3.3.2. Attention Mechanism

**Figure 4:**
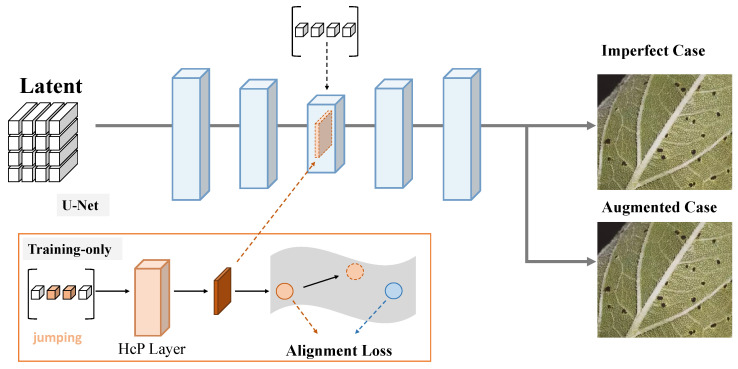
Schematic diagram of the attention mechanism module in disease detection.

The attention mechanism module plays a critical role in the network architecture, aiming to enhance the precision and efficiency of disease feature extraction, especially in scenarios involving complex backgrounds and subtle feature distributions. Its design is based on the multi-head attention (MHA) mechanism, which integrates global contextual information with local feature enhancement capabilities. As illustrated in Figure 4, this attention mechanism is specifically tailored to disease detection tasks, further optimizing multi-scale feature learning.

The proposed attention mechanism consists of three main submodules: query, key, and value matrix generation, the attention computation module, and the multi-head output fusion module. The input feature dimensions are set as width=64, height=64, and channels=256. After a convolutional layer, the features are mapped into the query, key, and value spaces, each with a dimensionality of channels=64, as defined by

(21) $$Q=W^{Q}\cdot X,\;K=W^{K}\cdot X,\;V=W^{V}\cdot X,$$

where $X\in R^{64\times 64\times 256}$ represents the input feature map, and $W^{Q}$, $W^{K}$, and $W^{V}$ are the weight matrices for mapping the features to query, key, and value spaces, respectively. The resulting matrices have dimensions of $R^{64\times 64\times 64}$. In the attention computation module, the query and key matrices are multiplied to generate an attention weight matrix, which is normalized to obtain the final attention distribution

(22) $$A=\mathrm{softmax}\left(\frac{Q\cdot K^{T}}{\sqrt{d_{k}}}\right),$$

where $A\in R^{64\times 64}$ is the attention matrix, and $d_{k}$ represents the dimension of the key matrix, used to scale the dot product to prevent numerical instability. Finally, the attention matrix is multiplied by the value matrix to generate the enhanced output features

(23) Z=A·V,

where $Z\in R^{64\times 64\times 64}$ represents the output features from the attention mechanism. The multi-head attention mechanism processes multiple independent attention heads in parallel, concatenating their outputs and applying a linear transformation to obtain the final result

(24) $$\mathrm{MHA}\left(Q,K,V\right)=\mathrm{Concat}\left({\mathrm{head}}_{1},\ldots ,{\mathrm{head}}_{h}\right)\cdot W^{O},$$

where ${\mathrm{head}}_{i}=A_{i}\cdot V_{i}$ represents the output of the *i*th attention head, and $W^{O}$ is the linear transformation matrix. The proposed design incorporates h=8 attention heads, each with a channel dimension of 64, resulting in a total output feature dimension of $R^{64\times 64\times 256}$. Mathematically, the core functionality of the attention mechanism is to compute a weighted sum of the input features, emphasizing the features relevant to the current task while suppressing redundant or irrelevant information. Let $X\in R^{N\times d}$ denote the input features, where *N* represents the number of feature points and *d* represents the feature dimension. Unlike traditional convolutional operations with fixed weights, the attention mechanism dynamically generates weights, enabling the model to adaptively focus on task-relevant regions. The normalized attention matrix *A* satisfies the following property:

(25) $$\sum_{j=1}^{N}A_{ij}=1,\;\forall i,$$

ensuring that the attention mechanism performs a globally normalized weighted sum, preventing the dominance of any single feature while maintaining global information integrity.The integration of the attention mechanism with the FSDDN is crucial in this study. The intermediate features ${\hat{Z}}_{t}$ generated by the diffusion network serve as the input to the attention mechanism, where the MHA module further enhances the feature representations. The output features ${\hat{Z}}_{t}^{\prime }$ from the attention mechanism are then combined with the denoised features from the diffusion network as follows:

(26) $${\hat{Z}}_{t}^{\prime }=\mathrm{MHA}\left({\hat{Z}}_{t}\right)+{\hat{Z}}_{t}.$$

This design not only strengthens the global dependency relationships among features but also captures complex disease patterns through multi-scale feature fusion. When combined with the diffusion network, the attention mechanism significantly improves the accuracy of disease detection in few-shot scenarios. Its dynamic weighting capability effectively addresses challenges such as uneven disease distribution and background interference, providing robust support for disease detection tasks.

#### 3.3.3. Diffusion Loss Function

The diffusion loss function serves as the core optimization component of the proposed FSDDN, designed to iteratively minimize the divergence between the generated distribution and the real distribution. This approach enhances the quality of disease feature generation, ultimately improving detection performance. The diffusion loss function differs fundamentally from traditional loss functions, such as mean squared error or cross-entropy loss, by leveraging a stepwise denoising process. It models the discrepancy between the generated and target distributions at each step, ensuring the production of high-quality features. In diffusion models, noisy features are progressively restored to target features $Z_{0}$ through a multi-step denoising process. The key to this process is minimizing the divergence between the real distribution $q(Z_{t-1}|Z_{t})$ and the model-predicted distribution $p_{\theta }\left(Z_{t-1}|Z_{t}\right)$. This divergence is measured using the Kullback–Leibler (KL) divergence, and the diffusion loss function is defined as

(27) $$L_{\mathrm{Diffusion}}=E_{q(Z_{0:T})}\left[\sum_{t=1}^{T}D_{\mathrm{KL}}\left(q\left(Z_{t-1}|Z_{t},Z_{0}\right)∥p_{\theta }\left(Z_{t-1}|Z_{t}\right)\right)\right],$$

where $q(Z_{t-1}|Z_{t},Z_{0})$ represents the real denoising distribution, $p_{\theta }\left(Z_{t-1}|Z_{t}\right)$ denotes the model-predicted denoising distribution, and $D_{\mathrm{KL}}$ quantifies the divergence between these distributions. In practical terms, $q(Z_{t-1}|Z_{t},Z_{0})$ is defined as

(28) $$q\left(Z_{t-1}|Z_{t},Z_{0}\right)=N\left(Z_{t-1};{\tilde{\mu }}_{t}\left(Z_{t},Z_{0}\right),{\tilde{\beta }}_{t}I\right),$$

where ${\tilde{\mu }}_{t}$ and ${\tilde{\beta }}_{t}$ are the mean and variance of the real distribution, parameterized by the diffusion process. In contrast, the predicted distribution $p_{\theta }\left(Z_{t-1}|Z_{t}\right)$ uses the mean and variance generated by the neural network

(29) $$p_{\theta }\left(Z_{t-1}|Z_{t}\right)=N\left(Z_{t-1};\mu_{\theta }\left(Z_{t},t\right),\Sigma_{\theta }\left(Z_{t},t\right)\right).$$

Minimizing $L_{\mathrm{Diffusion}}$ ensures that the denoising process is progressively optimized, enabling the generated features to closely approximate the real disease features. The overall goal of the diffusion loss function is to minimize the negative log-likelihood of the entire generation process

(30) $$L_{\mathrm{total}}=-\log p_{\theta }\left(Z_{0}\right),$$

which can be expanded as

(31) $$L_{\mathrm{total}}=E_{q(Z_{0:T})}\left[\sum_{t=1}^{T}D_{\mathrm{KL}}\left(q\left(Z_{t-1}|Z_{t},Z_{0}\right)∥p_{\theta }\left(Z_{t-1}|Z_{t}\right)\right)\right]+E_{q(Z_{0})}\left[-\log p\left(Z_{0}\right)\right].$$

By iteratively minimizing the KL divergence at each step, the predicted distribution is progressively aligned with the real target distribution throughout the entire generation process. Mathematically, this stepwise optimization is equivalent to minimizing the divergence over the entire probability space, ensuring diversity and accuracy in the generated results. In the FSDDN, the diffusion loss function is directly applied to the generated feature distribution $Z_{t}$, ensuring that the optimized features better adapt to disease detection tasks. The high-quality features generated through the diffusion process, ${\hat{Z}}_{0}$, are then passed to the attention mechanism and classification modules, further enhancing the precision of disease region detection

(32) $${\hat{Z}}_{0}={\mathrm{argmin}}_{\theta }L_{\mathrm{Diffusion}}.$$

This process not only improves the quality of the generated features but also enhances the model’s generalization capabilities in data-scarce scenarios. The generated features effectively supplement the original dataset, providing diverse and representative training data for the few-shot learning framework. Compared to traditional loss functions, the diffusion loss function offers several advantages. First, it optimizes the generation process step by step, enabling the model to adapt to the complexity and diversity of the disease features. Second, when integrated with the few-shot learning framework, the generated features significantly improve the model’s generalization capabilities, particularly in scenarios with limited data and complex backgrounds. Third, the mathematical properties of the diffusion loss function ensure the stability of the generation process, providing robust theoretical support for disease detection tasks. Experimental results demonstrate that this method achieves superior performance in high-precision disease detection, significantly outperforming traditional generative methods and loss function designs.

#### 3.3.4. Analysis of Synergistic Benefits of Combining Few-Shot Learning and Diffusion Models

In sunflower disease detection tasks, issues such as scarce data samples, complex and diverse disease features, and significant background interference are the main challenges limiting model performance. To address these problems, this paper proposes an innovative framework combining few-shot learning and diffusion models, which, through their synergistic effect, effectively tackles the aforementioned challenges from multiple dimensions. Firstly, few-shot learning, by constructing support and query sets, enables efficient learning of feature distributions between classes with very few samples, achieving accurate classification and detection. This method is particularly suitable for scenarios with scarce data samples, especially in agricultural disease detection tasks, where it is often difficult to collect a sufficient number of labeled samples in real-world field conditions. However, relying solely on few-shot learning may result in insufficient feature representation when faced with complex backgrounds and diverse disease features. For instance, when the background contains interfering features similar to the disease or when the disease area’s features are ambiguous, few-shot learning has limited capacity to capture these details. As a generative model, the diffusion model can generate high-quality latent feature distributions through a step-by-step restoration process. Its essence lies in learning the complexity of feature distributions guided by noise. Unlike traditional generative models, diffusion models can effectively model the multimodal characteristics of data during the generation process while enhancing the expression of fine-grained features through a stepwise denoising mechanism. Therefore, the diffusion model not only compensates for the deficiencies in feature diversity of few-shot learning but also provides more representative and robust feature descriptions for both the support and query sets. The synergistic benefits of combining these two techniques manifest in several aspects. First, the high-quality features generated by the diffusion model can enrich the feature representation of the support set, making the category prototypes in few-shot learning more accurate, thereby improving the classification performance for query samples. This synergistic effect is particularly significant in data-scarce scenarios, where the generated features of the diffusion model act as a “virtual supplement” to the data, providing few-shot learning with more diverse and representative training features. Second, few-shot learning can effectively leverage the features generated by the diffusion model and optimize the practical application of the features through the matching mechanism between the support and query sets. This combination ensures that the model can accurately distinguish subtle differences between different categories, thus significantly enhancing overall detection performance. Additionally, the diffusion model demonstrates significant advantages in handling complex backgrounds and the diversity of disease features. By generating feature distributions that contain rich details and global information, the diffusion model helps few-shot learning to better focus on key disease features while suppressing the influence of background interference. For example, in sunflower disease detection tasks, the background may contain noise information similar to disease patterns, such as leaf texture or non-disease spots. The features generated by the diffusion model can effectively enhance the model’s ability to discriminate disease regions through multimodal distribution modeling and noise suppression mechanisms.

### 3.4. Evaluation

To comprehensively evaluate the sunflower disease detection method proposed in this paper, based on few-shot learning and diffusion models, common performance metrics such as precision (P), recall (R), accuracy (Acc), and mean average precision at a 75% intersection-over-union (IoU) threshold (mAP@75) are employed. Precision measures the proportion of true positive samples among all samples predicted as positive, reflecting the model’s prediction accuracy. Recall indicates the proportion of actual positive samples that are correctly identified as positive by the model, showcasing the model’s detection capability. Accuracy, as a straightforward metric, evaluates the ratio of correct predictions across all predictions, suitable for initial overall performance assessments. Meanwhile, mAP is a crucial metric in object detection, assessing the model’s performance in multi-object and multi-class scenarios by calculating the average detection precision at different thresholds. Specifically, mAP@75 refers to the average detection precision at an IoU threshold of 0.75. First, sunflower disease detection tasks often require precise localization of disease-affected areas to ensure the reliability of diagnostic results and the accuracy of subsequent prevention and control measures. In practical agricultural scenarios, symptoms of sunflower diseases typically manifest as small spots, mildew layers, or localized yellowing, which demand the model to provide highly accurate bounding box annotations for detection. Using a lower IoU threshold (e.g., 0.50) could result in the model showing some degree of inaccuracy in bounding box localization, thereby affecting its practical utility. By enforcing a higher IoU requirement, mAP@75 encourages the model to produce bounding boxes that closely align with the actual distribution of disease-affected areas, thus improving the overall detection accuracy of sunflower diseases. These metrics enable a comprehensive evaluation of the model’s performance from different perspectives, providing clear directions for optimizing disease detection methods. The formula for precision (*P*) is provided by

(33) $$P=\frac{TP}{TP+FP},$$

where TP denotes the number of true positives (i.e., correctly predicted positive samples) and FP denotes the number of false positives (i.e., incorrectly predicted as positive samples). The formula for recall (*R*) is

(34) $$R=\frac{TP}{TP+FN},$$

where FN represents the number of false negatives (i.e., positive samples that were incorrectly predicted as negative). Recall measures the model’s detection ability, particularly in disease detection tasks, where a higher recall ensures that as many disease samples as possible are detected. Acc reflects the overall correctness of the model’s predictions, and its formula is

(35) $$Acc=\frac{TP+TN}{TP+TN+FP+FN},$$

where TN represents the number of true negatives (i.e., correctly predicted negative samples). Accuracy provides an intuitive reflection of the model’s overall performance, suitable for evaluating the model’s general capabilities across various scenarios. The computation of mean average precision (mAP) is based on the average precision (AP) for each class. To calculate AP, precision and recall are computed for each IoU threshold, followed by interpolation to determine the average value. For a specific class c and threshold t, the formula for AP is

(36) $$AP_{c}=\int_{0}^{1}P\left(R\right)\;dR,$$

where P(R) is the precision–recall curve. The mAP is the average of the AP values across all classes

(37) $$mAP=\frac{1}{C}\sum_{c=1}^{C}AP_{c},$$

where *C* denotes the total number of classes. The mAP@75 specifically refers to the average detection precision at an IoU threshold of 0.75, used to measure the model’s performance in high-precision tasks. Through the comprehensive evaluation of these metrics, the model’s performance in sunflower disease detection tasks can be thoroughly understood, reflecting both its robustness in complex scenarios and providing guidance for optimizations in different application contexts. For instance, in practical disease detection, recall may be prioritized to ensure that important disease samples are not missed, while, in disease classification or grading tasks, precision becomes more critical. The mAP metric is particularly significant as it provides a more comprehensive performance evaluation in multi-object and multi-class detection scenarios. The F1-score is a harmonic mean of precision and recall, providing a balanced measure of a model’s performance by considering both false positives and false negatives. It is particularly useful in evaluating imbalanced datasets where one class may dominate the others. The F1-score is defined as

(38) $$F1=2\cdot \frac{\mathrm{Precision}\cdot \mathrm{Recall}}{\mathrm{Precision}+\mathrm{Recall}}$$

By combining these metrics, a more accurate comparison of the proposed method with existing baseline models is facilitated, thus verifying the effectiveness of the proposed approach.

### 3.5. Baseline

To comprehensively assess the performance of the sunflower disease detection method proposed in this paper, based on few-shot learning and diffusion models, several representative models from the fields of object detection and disease detection were selected as baselines for comparison experiments. These models include RT-DETRv2 [38], Mask R-CNN [39], LeafDetection [6], and DETR [40], all of which have demonstrated excellent detection capabilities in various scenarios. RT-DETRv2 is an efficient real-time object detection model that features low latency and high accuracy, making it particularly suitable for real-time detection tasks. Mask R-CNN is a classic instance segmentation model that not only detects object boundaries but also precisely segments disease areas, providing rich pixel-level feature information for complex scenarios. LeafDetection is a model specifically designed for plant leaf disease detection, offering an efficient solution to the diversity and complexity of plant diseases. DETR (Deformable Transformer for End-to-End Object Detection) employs an innovative transformer-based detection framework that models global context information to achieve superior detection performance. These baseline models provide different dimensions of comparison for disease detection tasks. By comparing their performance with that of the proposed method, the effectiveness of diffusion models and few-shot learning in disease detection tasks can be more clearly verified. All comparison experiments are conducted using the same dataset split and evaluation metrics, including precision, recall, accuracy, and mAP@75, to ensure fairness and comparability of the results.

### 3.6. Hardware and Software Platforms

The experimental environment was set up on a high-performance hardware platform to ensure the efficiency and stability of model training and inference. The primary hardware device is the NVIDIA A100 GPU, a graphics card designed for high-performance computing and deep learning tasks. It features 80 GB of VRAM, supports mixed-precision computation, and enables large-scale parallel processing, effectively handling the complex computational demands of neural networks. The server housing the GPU is equipped with dual Intel Xeon processors, providing robust central processing and data transfer capabilities. Additionally, high-speed NVMe solid-state drives (SSDs) were used for quick data loading and model weight storage. The server also contains at least 256 GB of memory, ensuring ample memory space for training and inference of large-scale deep learning models, thereby avoiding bottlenecks due to insufficient memory.

The software platform is primarily based on the PyTorch framework, a flexible and powerful deep learning library that supports dynamic computation graphs and large-scale distributed training, making it ideal for the complex model development requirements of this experiment. The operating system is Ubuntu 20.04 LTS, a Linux-based platform providing a stable runtime environment and robust command-line tool support. For data processing, Python 3.8 and its extensive ecosystem are employed, including OpenCV 4.0 for image preprocessing and visualization, Numpy 1.21.0 for numerical computation, and Pandas for statistical analysis. To implement the diffusion model for training and inference, the HuggingFace Diffusers 0.26.0 library was used. This library offers an efficient and flexible implementation of diffusion models, supporting various diffusion model architectures and facilitating rapid experimentation and model fine-tuning. These tools collectively form the software foundation of the experiment, ensuring the efficient execution of the disease detection tasks.

### 3.7. Optimizer and Hyperparameter Settings

The AdamW optimizer was employed in this study due to its superior performance in deep learning tasks and its ability to effectively mitigate the issue of weight decay during model training. AdamW dynamically adjusts the learning rate for each parameter, achieving more stable convergence. The initial learning rate was set to η=0.0002, a value that has been shown in experiments to balance the trade-off between convergence speed and model performance. In addition, to accelerate model training and improve gradient computation efficiency, the batch size was set to 32. This setting maximizes the parallel computing capabilities of the GPU while preventing out-of-memory errors due to excessive memory usage.

The dataset was split into training, validation, and test sets in an 8:1:1 ratio, ensuring that 80% of the data were used for training, 10% for validation, and 10% for testing. This division enables the model to learn effectively during training, fine-tune hyperparameters using the validation set, and evaluate final performance on the test set. To further enhance the model’s robustness and generalization, 5-fold cross-validation was introduced. The training set was divided into 5 non-overlapping subsets, with 4 subsets used for training and the remaining subset used for validation in each fold. Cross-validation helps to alleviate the performance fluctuations caused by data partitioning differences, providing a more comprehensive evaluation of the model’s capabilities.

In training the diffusion model, the noise steps *T* were set to 1000, a critical hyperparameter that controls the refinement process of recovering the data distribution from pure Gaussian noise. A higher number of noise steps generates more refined and higher-quality samples while maintaining training stability. Other important hyperparameters were also fine-tuned, such as the weight decay coefficient $\lambda_{wd}=0.01$, which controls the model complexity and prevents overfitting. These settings provided a stable training environment and optimization strategy, ensuring the model’s exceptional performance in sunflower disease detection tasks.

## 4. Results and Discussion

### 4.1. Disease Detection Results

The objective of this experiment was to evaluate the performance of various models in sunflower disease detection tasks by comparing four key metrics: precision, recall, accuracy, F1-score, and mean average precision (mAP@75). The results demonstrate that model performance improved significantly with architectural optimization and task-specific adjustments, particularly in challenging scenarios involving complex backgrounds and limited sample sizes. As shown in Table 2, RT-DETRv2 exhibited the lowest performance across all four metrics, mainly due to its focus on global features, while lacking the ability to capture fine details. Mask R-CNN achieved better results through its instance segmentation approach, but its segmentation accuracy for small disease spots remained inadequate. The DETR model, leveraging a transformer-based architecture, showed strong overall performance but still faced challenges in handling background noise and detecting small disease regions. The LeafDetection model, specifically designed for sunflower disease detection, significantly enhanced fine-grained feature extraction, making it advantageous for identifying small disease spots. However, the proposed method outperformed all the other models across all the metrics, achieving a precision of 0.94, recall of 0.92, accuracy of 0.93, F1-score of 0.93, and mAP@75 of 0.92. This indicates that the integration of advanced deep learning techniques and customized disease-specific feature modeling enables highly efficient disease detection in complex agricultural scenarios.

From a theoretical perspective, the performance of these models is closely tied to their mathematical structures and design principles. RT-DETRv2 relies on self-attention mechanisms to capture global features, excelling in scenarios with long-range dependencies. However, it lacks task-specific optimizations for disease detection, resulting in insufficient support for detailed feature extraction. Mask R-CNN combines an RPN with an instance segmentation module, enhancing its ability to process object boundaries in complex backgrounds. Nevertheless, its CNN-based structure struggles to integrate global information effectively, particularly when the contrast between disease spots and the background is weak, leading to potential misclassifications. The DETR model, adopting an end-to-end transformer architecture, leverages global contextual information to achieve high detection precision. However, its limited focus on small targets could result in missed detections for minor disease spots. The LeafDetection model, with its convolutional layers specifically designed to model disease features, improves segmentation accuracy for small disease regions but does not fully exploit multi-scale global information. The proposed method integrates the strengths of these approaches, employing multi-head self-attention mechanisms to enhance global contextual modeling while leveraging CNNs for local feature extraction. Additionally, disease-specific customizations were introduced, enabling the model to excel in both complex backgrounds and fine-grained feature recognition. The experimental results validate that this combined strategy effectively addresses the limitations of conventional models, advancing the application of deep learning in agricultural disease detection.

### 4.2. Experimental Results for Different Disease Types

This experiment aimed to evaluate the performance of the proposed detection method across different types of diseases and analyze its generalization capability and adaptability in detection tasks. As shown in Table 3, the model demonstrated high performance across all five disease types, although variations were observed in detection effectiveness. For black spot disease, a precision of 0.91, recall of 0.88, and mAP@75 of 0.90 were achieved, indicating strong performance in capturing primary disease features. However, a slightly higher miss rate may have occurred due to the randomness of the disease spots and the complexity of the background. The detection performance for brown spot disease further improved, with precision and recall reaching 0.93 and 0.91, respectively, and an mAP@75 of 0.91, suggesting that the model effectively captured the distinctive color and boundary features of brown spot disease. Downy mildew achieved precision and recall of 0.94 and 0.92, respectively, reflecting the model’s ability to capture the subtle features of the mildew distribution on the leaf underside, demonstrating robust modeling of complex disease characteristics. The highest performance was observed for wilt disease and rust disease, with precision values of 0.96 and 0.97 and recall values of 0.94 and 0.93, respectively. These results highlight the model’s significant advantage in feature extraction and classification for these diseases, particularly in accurately identifying the shapes and distributions of rust spots on leaves.

From a theoretical perspective, the differences in detection performance across disease types are closely related to the complexity of their features and distribution patterns. The relatively lower detection precision for black spot disease and brown spot disease may stem from the variability in spot morphology and color under different environmental conditions, requiring the model to adapt to more complex distributions during feature learning. The higher detection performance for downy mildew can be attributed to the strong local consistency of its mildew layer characteristics, which the model effectively captured through attention mechanisms and multi-scale feature extraction. Additionally, wilt disease and rust disease achieved the best detection performance due to their relatively concentrated regional distributions and prominent features. The model leveraged global context modeling and convolutional operations to accurately extract the critical patterns of these diseases. Mathematically, the proposed method generates high-quality latent features using a diffusion model, which, when combined with the few-shot learning framework, constructs more robust class prototypes between the support set and query set. This enhances the clarity of inter-class feature differences. The attention mechanism further optimizes the representation of disease regions, significantly improving the model’s robustness in complex backgrounds. These designs enable the model to capture feature distribution differences on a global scale while enhancing its focus on boundaries and subtle features at a local level. As demonstrated by the experimental results, the proposed method exhibits outstanding detection capabilities across various disease types, offering substantial advantages for intelligent agricultural disease detection in complex scenarios.

### 4.3. Ablation Study on Different Generation Methods

This experiment aimed to investigate the impact of different generation methods on disease detection tasks by comparing the performance improvements achieved through AutoEncoder, GANs, and diffusion models. As shown in Table 4, the ablation study validated the pivotal role of the diffusion model in the proposed method. The experimental results demonstrated significant performance differences among the three generation methods, with the diffusion model outperforming the others. Specifically, the precision, recall, accuracy, and mAP@75 of AutoEncoder were 0.73, 0.70, 0.72, and 0.71, respectively, indicating limited capability in generating disease features. The GAN showed substantial improvements over AutoEncoder, achieving 0.85, 0.81, 0.83, and 0.82 across the same metrics, reflecting its potential in capturing complex feature distributions. However, the diffusion model achieved the best results across all the metrics, with precision reaching 0.94, recall 0.90, accuracy 0.91, and mAP@75 0.92, firmly establishing its applicability in generating high-quality features, particularly under small-sample scenarios.

From a theoretical perspective, the differences in performance among these generation methods can be attributed to their mathematical characteristics and intrinsic mechanisms for feature generation. AutoEncoder, employing an encoder–decoder structure to compress and reconstruct input features, is capable of generating basic semantic features. However, its linear mapping approach limits its ability to model complex distributions and local details. A GAN introduces nonlinear mapping through adversarial learning between the generator and discriminator, aiming to approximate real distributions. Despite their ability to capture more intricate features, GANs are prone to mode collapse during training, resulting in insufficient diversity in the generated samples. In contrast, the diffusion model employs a probabilistic generation framework based on a stepwise denoising process. Starting from random Gaussian noise, it iteratively learns the reverse diffusion process to restore the target distribution. Its loss function, grounded in KL divergence, aims to minimize the discrepancy between the generated and true distributions at each step

(39) $$L_{\mathrm{Diffusion}}=E_{q(Z_{0:T})}\left[\sum_{t=1}^{T}D_{\mathrm{KL}}\left(q\left(Z_{t-1}|Z_{t},Z_{0}\right)∥p_{\theta }\left(Z_{t-1}|Z_{t}\right)\right)\right],$$

This iterative optimization strategy ensures both the quality and diversity of the generated features. Moreover, the multi-step denoising mechanism enhances the modeling of complex backgrounds and disease characteristics, producing refined and representative features. This makes the diffusion model particularly suitable for disease detection tasks involving scarce data and intricate backgrounds. The experimental results confirm, both theoretically and empirically, that the diffusion model surpasses its counterparts in feature generation quality, distribution richness, and task adaptability. These advantages significantly contribute to the superior performance of the proposed method in disease detection, providing robust support for its high accuracy and reliability.

### 4.4. Ablation Study on Different Loss Functions

This experiment was designed to investigate the impact of different loss functions on disease detection tasks. By comparing the performance of cross-entropy loss, focal loss, and diffusion loss, the optimization effects of these loss functions on model performance were analyzed. As shown in Table 5, the experimental results demonstrated that diffusion loss significantly outperformed the other two loss functions across all the evaluation metrics, achieving a precision of 0.94, recall of 0.90, accuracy of 0.91, and mAP@75 of 0.92. In comparison, focal loss performed moderately well, with a precision of 0.83, recall of 0.80, accuracy of 0.81, and mAP@75 of 0.81. Cross-entropy loss exhibited the weakest performance, with all the metrics below 0.70. These findings indicate that the choice in loss function is critical to the performance of disease detection tasks, particularly in scenarios involving small samples and complex backgrounds. Diffusion loss demonstrated its advantage in enhancing the feature generation quality and improving the model’s generalization ability.

From a theoretical perspective, the performance differences among these loss functions can be attributed to their mathematical formulations and optimization objectives. Cross-entropy loss is designed to optimize the log-likelihood for classification tasks, making it suitable for scenarios with balanced class distributions. However, when confronted with imbalanced data and small sample challenges, it is prone to gradient updates biased toward dominant classes, thereby reducing sensitivity to small disease regions. Focal loss, by introducing modulation factors α and γ, applies weighted gradients to hard-to-classify samples, with its optimization objective defined as

(40) $$L_{\mathrm{Focal}}=-\alpha {\left(1-p_{t}\right)}^{\gamma }\log\left(p_{t}\right),$$

where $p_{t}$ represents the predicted probability for the correct class, and α and γ control the weight distribution for sample difficulty. This design enhances learning for small samples and hard-to-classify examples, outperforming cross-entropy loss in imbalanced scenarios. However, its optimization remains focused on single-step classification probabilities, lacking the ability to model global feature distributions, which limits its adaptability to complex backgrounds and multimodal disease features. In contrast, diffusion loss is designed from a generative model perspective, with the objective of progressively minimizing the divergence between generated and true distributions. Based on KL divergence, it is defined as

(41) $$L_{\mathrm{Diffusion}}=E_{q(Z_{0:T})}\left[\sum_{t=1}^{T}D_{\mathrm{KL}}\left(q\left(Z_{t-1}|Z_{t},Z_{0}\right)∥p_{\theta }\left(Z_{t-1}|Z_{t}\right)\right)\right],$$

Through a multi-step optimization process, the model is capable of generating high-quality disease features while capturing complex distribution patterns and fine-grained details. Mathematically, the diffusion loss function’s optimization of each step’s generation results corresponds to the global modeling of the entire generation trajectory, ensuring the robustness of the generated features in multi-scale and complex backgrounds. This characteristic is particularly crucial in disease detection tasks, where background noise interference and diverse disease features must be effectively addressed. Diffusion loss enhances feature representation significantly, improving detection performance. The experimental results validate these theoretical insights. Diffusion loss, through its progressive optimization strategy, exhibits superior adaptability and accuracy in complex agricultural scenarios. It provides strong theoretical support and practical value for advancing high-performance disease detection tasks.

### 4.5. Tasks on Other Datasets

In addition to the experiments conducted on the primary dataset, an independent dataset was also tested to further validate the generalization performance and robustness of the proposed method. This dataset contains four distinct categories of leaf images: downy mildew, fresh leaf, gray mold, and leaf scars. The numbers of images in each category are 120, 134, 72, and 140, respectively. The diversity of this dataset is reflected in the significant differences in disease types and leaf conditions, especially for categories such as downy mildew and gray mold, which exhibit complex characteristics, indistinct boundaries, and are prone to interference from the background during detection. To ensure experimental consistency, the annotation process for this dataset followed the same approach as that of the primary dataset, employing precise bounding box annotations that were verified by experts with experience in agricultural disease detection. The annotation process focused on key features such as the shape, size, and color of the disease regions to ensure that the model could effectively capture disease-specific characteristics during training. Additionally, to mitigate training biases caused by class imbalance, data balancing techniques were applied during training, including data augmentation operations such as rotation, cropping, and color jittering. As shown in Table 6, these augmentations expanded the dataset and improved the robustness of the model.

The objective of this experiment was to validate the generalization performance and robustness of the proposed method on an independent dataset. Compared to the primary dataset, this dataset exhibits greater diversity in leaf conditions and background complexity. Through the experiments, the adaptability of the proposed framework, which integrates few-shot learning and diffusion models, to various disease feature distributions was analyzed. Additionally, by comparing its performance with several baseline models, the effectiveness of the proposed method in precise detection tasks was validated. The proposed method outperformed the other models across every metric, achieving a precision of 0.92, recall of 0.90, accuracy of 0.91, and mAP@75 of 0.91. These results demonstrate that the proposed method can achieve high detection accuracy and robustness in scenarios with complex backgrounds and diverse feature distributions. In contrast, traditional methods such as RT-DETRv2 and Mask R-CNN, while capable of handling simple disease features to some extent, showed significant limitations in scenarios with complex backgrounds or blurred features. From a theoretical perspective, the performance of each model is closely related to its structural design. RT-DETRv2, which is based on the transformer architecture, demonstrates strong global feature modeling capabilities and can handle long-range dependencies. However, its lack of optimization for small disease regions leads to suboptimal performance in detecting diseases with indistinct boundaries. Mask R-CNN, which combines a region proposal network (RPN) with instance segmentation techniques, performs well in multi-object scenarios. However, its convolutional operations focus primarily on local features, lacking the ability to model global information effectively, resulting in potential misdetections or missed detections in scenarios with significant background interference. Moreover, DETR employs an end-to-end transformer architecture, leveraging global contextual relationships to enhance detection accuracy, but its capacity for learning feature distributions in few-shot disease cases is limited. The LeafDetection model was specifically optimized for sunflower disease detection, and its convolutional layers are well-suited for extracting small disease region features. However, its generalization capability is somewhat constrained.

**Figure 5:**
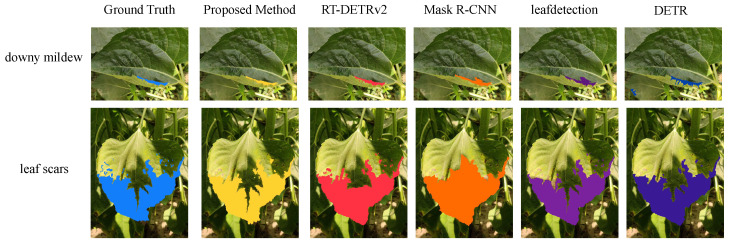
Visualization analysis of the experimental results.

In comparison, the proposed method combines high-quality features generated by the diffusion model with the feature extraction capabilities of few-shot learning. By incorporating attention mechanisms and the diffusion loss function, the proposed method significantly enhances the expression of disease features and improves its adaptability to different disease types. This design better balances the requirements of global feature modeling and local detail extraction, effectively addressing the challenges posed by data scarcity and background complexity. As a result, the proposed method demonstrated outstanding performance across various disease detection tasks. The experimental results validate the robustness and superiority of the proposed method from both mathematical design and practical application perspectives, providing robust technical support for agricultural disease detection tasks. Based on the experimental results, a visualization analysis was conducted, as shown in Figure 5.

### 4.6. Deployment in Bayannur

The method proposed in this study not only presents an effective solution to the problem of data scarcity at the theoretical level but has also been validated and deployed in practical agricultural scenarios. The method has preliminarily been deployed in sunflower cultivation fields in Bayannur, Inner Mongolia, China, specifically for sunflower disease detection and prevention tasks. During the field testing phase, the system was deployed in multiple sunflower cultivation plots, with high-altitude images captured by drones. Combined with the rapid computational capabilities of edge devices, precise disease detection was achieved. The system’s detection results were uploaded to the cloud platform via wireless networks for analysis and decision-making by agricultural technicians. After a period of actual operation, the model achieved a precision of 0.94, recall of 0.92, accuracy of 0.93, and mAP@75 of 0.92, which were consistent with the laboratory test results. Based on the disease distribution information detected by the system, agricultural technicians were able to quickly formulate disease control strategies, such as pesticide spraying and adjusting planting density, thus significantly improving the disease prevention efficiency and reducing the labor costs. Furthermore, the diversified features generated by the diffusion model helped the system to effectively separate disease from background in complex field scenarios, enabling it to adapt to the data-scarce and environmentally complex agricultural scenes in Bayannur. This provides a practical solution to the data scarcity problem in agricultural scenarios. The system has received unanimous praise from both agricultural technicians and farmers regarding the real-world deployment in Bayannur, Inner Mongolia. Farmers reported that, based on the detection results provided by the system, they were able to quickly take appropriate control measures, significantly reducing pesticide usage and control costs while improving crop yield and quality.

### 4.7. Limitation and Future Work

The proposed method has demonstrated outstanding performance across multiple experiments, particularly in the challenging task of sunflower disease detection. However, there are certain limitations that require further optimization and improvement. First, regarding the dependency on high-performance computational resources, the method employs a diffusion model during the training process. This model generates high-quality features through a multi-step denoising process, significantly enhancing the accuracy and robustness of disease detection. One of the core characteristics of diffusion models is their iterative optimization process, which requires substantial computational resources. Specifically, in this study, the number of diffusion steps (TTT) was set to 1000, with each step involving feature generation and optimization. As a result, the training time and resource consumption are significantly higher compared to the traditional methods. In the experiments, training was conducted on an NVIDIA A100 GPU (80 GB memory), requiring approximately 72 h for a complete training cycle. If lower-performance hardware (e.g., NVIDIA 1080Ti or RTX 3060) were used, the training time could double or even increase further. Additionally, the multi-step computation process of the diffusion model imposes high memory requirements. For instance, training on high-resolution images requires more than 48 GB of GPU memory. This reliance on high-performance computational resources somewhat limits the practical applications of the method, especially in resource-constrained agricultural environments, such as edge devices or low-cost hardware. Future work could address this limitation by designing more efficient generative models, such as lightweight diffusion models, or by leveraging techniques like knowledge distillation to reduce the model size and computational demands.

## 5. Conclusions

In recent years, agricultural disease detection tasks have faced numerous challenges, including data scarcity, complex and diverse disease characteristics, and significant background interference. To address these issues, the proposed method integrated the feature extraction capabilities of few-shot learning with the high-quality feature generation ability of diffusion models, forming an end-to-end disease detection framework. By designing the FSDDN, the method achieved progressive restoration from noisy features to high-quality disease features. Additionally, attention mechanisms were incorporated to optimize the disease feature representations, and a diffusion loss function tailored to the distribution of the disease features was introduced. The experimental results demonstrate the outstanding performance of the proposed method across multiple evaluation metrics. For instance, in the overall disease detection task, the proposed method achieved precision, recall, accuracy, and mAP@75 scores of 0.94, 0.92, 0.93, and 0.92, respectively, significantly surpassing the other comparative models. In the ablation experiments for the different generation methods, the diffusion model outperformed the AutoEncoder and GAN models across all the evaluation metrics. Similarly, in the ablation experiments for the loss functions, the diffusion loss function outperformed focal loss by 11%, 10%, 10%, and 11% in precision, recall, accuracy, and mAP@75, respectively, and exhibited even greater performance improvements compared to traditional cross-entropy loss. Regarding the experimental results for the different disease types, the proposed method demonstrated excellent performance in detecting black spot disease, brown spot disease, downy mildew, wilt disease, and rust disease. Particularly, in detecting wilt disease and rust disease, precision reached 0.96 and 0.97, while recall achieved 0.94 and 0.93, respectively, showcasing the method’s strong adaptability to specific disease types. The primary innovations and contributions of this study are as follows. First, a novel framework for disease detection based on few-shot learning and diffusion generative models was proposed. Second, a diffusion loss function was designed to leverage the multi-step optimization characteristics of the generative model, enhancing the model’s ability to learn complex feature distributions. Finally, the integration of attention mechanisms significantly improved the quality of the disease feature representations and enhanced the model’s capability to capture fine-grained features.

## Figures and Tables

**Table 1:** Data quantities for different sunflower diseases.

Disease	Data
black spot disease	1002
brown spot disease	1803
downy mildew	1947
wilt disease	1533
rust disease	1768

**Table 2:** Experimental results of disease detection models.

Model	Precision	Recall	Accuracy	mAP@75	F1-Score
RT-DETRv2	0.83	0.80	0.82	0.81	0.81
Mask R-CNN	0.87	0.84	0.86	0.85	0.85
DETR	0.89	0.86	0.87	0.87	0.87
LeafDetection [6]	0.92	0.89	0.91	0.90	0.90
Proposed Method	0.94	0.92	0.93	0.92	0.93

**Table 3:** Experimental results for different disease types.

Disease Type	Precision	Recall	Accuracy	mAP@75	F1-Score
black spot disease	0.91	0.88	0.89	0.90	0.89
brown spot disease	0.93	0.91	0.92	0.91	0.92
downy mildew	0.94	0.92	0.93	0.93	0.93
wilt disease	0.96	0.94	0.95	0.94	0.95
rust disease	0.97	0.93	0.95	0.94	0.95

**Table 4:** Ablation study of different generation methods.

Disease Type	Precision	Recall	Accuracy	mAP@75	F1-Score
AutoEncoder	0.73	0.70	0.72	0.71	0.71
GAN	0.85	0.81	0.83	0.82	0.83
Diffusion	0.94	0.92	0.93	0.92	0.93

**Table 5:** Ablation study of different loss functions.

Loss Function	Precision	Recall	Accuracy	mAP@75	F1-Score
Cross-Entropy Loss	0.69	0.65	0.67	0.67	0.67
Focal Loss	0.83	0.80	0.81	0.81	0.81
Diffusion Loss	0.94	0.92	0.93	0.92	0.93

**Table 6:** Experimental results of disease detection models on other datasets.

Model	Precision	Recall	Accuracy	mAP@75	F1-Score
RT-DETRv2	0.84	0.80	0.82	0.81	0.82
Mask R-CNN	0.86	0.82	0.84	0.83	0.84
DETR	0.87	0.85	0.86	0.86	0.86
LeafDetection [6]	0.90	0.89	0.90	0.90	0.89
Proposed Method	0.92	0.90	0.91	0.91	0.91

## Data Availability

The data presented in this study are available on request from the corresponding author.
